# Versatile vacuum-powered artificial muscles through replaceable external reinforcements

**DOI:** 10.3389/frobt.2023.1289074

**Published:** 2024-01-04

**Authors:** Mijaíl Jaén Mendoza, Sergio Cancán, Steve Surichaqui, Esteban Centeno, Ricardo Vilchez, Katia Bertoldi, Emir A. Vela

**Affiliations:** ^1^ Department of Mechanical Engineering, Universidad de Ingenieria y Tecnologia - UTEC, Lima, Peru; ^2^ Department of Mechatronics Engineering, Universidad de Ingenieria y Tecnologia - UTEC, Lima, Peru; ^3^ Department of Bioengineering, Universidad de Ingenieria y Tecnologia - UTEC, Lima, Peru; ^4^ J. A. Paulson School of Engineering and Applied Sciences, Harvard University, Cambridge, MA, United States; ^5^ Research Center in Bioengineering, Universidad de Ingenieria y Tecnologia - UTEC, Lima, Peru

**Keywords:** artificial muscle, reusable, versatile, soft actuator, replaceable reinforcements, soft robotics

## Abstract

Soft pneumatic artificial muscles are a well actuation scheme in soft robotics due to its key features for robotic machines being safe, lightweight, and conformable. In this work, we present a versatile vacuum-powered artificial muscle (VPAM) with manually tunable output motion. We developed an artificial muscle that consists of a stack of air chambers that can use replaceable external reinforcements. Different modes of operation are achieved by assembling different reinforcements that constrain the output motion of the actuator during actuation. We designed replaceable external reinforcements to produce single motions such as twisting, bending, shearing and rotary. We then conducted a deformation and lifting force characterization for these motions. We demonstrated sophisticated motions and reusability of the artificial muscle in two soft machines with different modes of locomotion. Our results show that our VPAM is reusable and versatile producing a variety and sophisticated output motions if needed. This key feature specially benefits unpredicted workspaces that require a soft actuator that can be adjusted for other tasks. Our scheme has the potential to offer new strategies for locomotion in machines for underwater or terrestrial operation, and wearable devices with different modes of operation.

## 1 Introduction

Introducing flexible materials strengthened the development of pneumatic actuation schemes, establishing a new paradigm in robotics. Such materials include mainly elastomers and fabrics that are commonly used in soft pneumatic actuators. This type of pneumatic actuation scheme offers key features like compliance and low-cost fabrication for robotic operation systems. As a result, soft actuators have been used extensively for wearable assistive devices (Roche et al., 2014; Kulasekera et al., 2020; Nguyen and Zhang, 2020; Thalman and Artemiadis, 2020; Mendoza et al., 2021) and grasping of objects (Miron et al., 2018; Fatahillah et al., 2020). One of the most known schemes in soft pneumatic actuators are Pneumatic Artificial Muscles (PAMs), offering simplicity and high force output for soft machines. PAMs have typically focused on achieving large contraction ratios (Hawkes et al., 2016; Li et al., 2017; Felt et al., 2018; Usevitch et al., 2018), and recent efforts have successfully replicated biological muscle performance in terms of stress and strain (Yang et al., 2016). Although biological systems have inspired the development of PAMs that exhibit a wide range of single type of movements (e.g., twisting, bending, rotary, etc.) (Niiyama et al., 2014; Polygerinos et al., 2015; Rus and Tolley, 2015; Robertson and Paik, 2017; Yang et al., 2017; Jiao et al., 2021; Joe et al., 2021), most PAMs are unable to produce more than two single type or complex type of movements, posing limitations to practical engineering applications.

In PAMs, simplicity is strongly associated with preprogrammed motions that are embedded in the actuator throughout their fabrication. Using a simple design can produce a complex motion, involving an infinite degree of freedom. The pre-defined configuration between air chambers and reinforcements performs a specific and sophisticated motion. Yet the versatility of PAMs is limited to properly work in a single scenario as PAMs components (air chambers and reinforcements) are coupled permanently in fabrication techniques such as heat sealing and multi-step molding or in general assembly of the actuator (Galloway et al., 2013; Mosadegh et al., 2014; Li et al., 2017; Nguyen and Zhang, 2020; Jiao et al., 2021), disabling any possibility to modify the output motion after fabrication.

PAMs present a fixed morphology that consist of an internal air bladder (air chamber) with an external braid (reinforcement) to perform linear contraction upon pressurization (relative to ambient) (Chou and Hannaford, 1996; Daerden and Lefeber, 2001; Hawkes et al., 2016). Bending and twisting motion also rely on permanent assembly and can be produced through structural asymmetry along the actuator’s length, coupling reinforcements internally [strain limiting layers and air chambers geometry (Mosadegh et al., 2014; Wang et al., 2018; Bhat and Yeow, 2020)] or externally [fibers, sleeves or rigid frames (Polygerinos et al., 2015; Miron et al., 2018; Zhang et al., 2020)]. In contrast to pressurization in PAMs, there are vacuum-based actuators called Vacuum-powered Artificial Muscles (VPAMs). These actuators shrink by applying negative pressure (relative to ambient), reducing the risk of explosive failure (Hawkes et al., 2016; Li et al., 2017; Felt et al., 2018; Usevitch et al., 2018), and offering compactness for space-limited scenarios (Yang et al., 2016; Li et al., 2017; Robertson and Paik, 2017; Felt et al., 2018; Tawk et al., 2018; Usevitch et al., 2018; Mendoza et al., 2021). VPAMs can also produce linear contraction, and can be classified in Bellow Vacuum Actuators (BVAs) that use equidistant internal rings (Felt et al., 2018; Lee and Rodrigue, 2018), and Fluid-driven origami artificial muscles (FOAMs) that use contractile internal structures [typically a origami skeleton (Li et al., 2017; Mendoza et al., 2021), a spring (Kulasekera et al., 2020; Kulasekera et al., 2021), or a deployable structure (Yu et al., 2021)]. Additionally, a few rubber-based vacuum actuators were proposed using buckling elastomeric structures (Yang et al., 2015; Yang et al., 2016; Yang et al., 2017). Further, vacuum actuators can generate bending motion using embedded layers (Felt et al., 2018; Lin et al., 2020) and twisting motion through helix reinforcements (Jiao et al., 2019; Jiao et al., 2021). Although PAMs and VPAMs work for specific scenarios since they are associated only to a specific mode of operation (contraction, bending, twisting, etc.), any post-assembly modification in an artificial muscle is desirable, as it can modify the default output motion increasing their versatility.

For scenarios that evolve throughout operation, a reusable and versatile artificial muscle is desired to perform various tasks, as opposed to having to fabricate a new specific actuator for each task or function needed. To this end, some research groups have focused their efforts on modifying the default motion of actuators through passive and active mechanisms, enabling mechanical programming after fabrication. However, these attempts are limited to manually change actuator’s length (Gollob et al., 2022) or to modify the curvature radius in bending actuators such as replacing components of the soft pneumatic actuators as chambers (Natividad et al., 2018), and reinforcements [flexible sleeves (Galloway et al., 2013) and rigid shells (Chen et al., 2017)], as well as embedding smart materials as internal layers (Firouzeh et al., 2015; Yoshida et al., 2018). A few studies have demonstrated promising results, creating multifunctional artificial muscles capable of producing omnidirectional bending (Murali Babu et al., 2023), bending and pure contraction (Lin et al., 2020) as well as 7 modes of operation (Zhang et al., 2023). These efforts consist of a single or stack of modular chambers that requires an additional system for automatable reconfiguration [fluidic channels (Murali Babu et al., 2023), layer jamming (Felt et al., 2018; Lin et al., 2020) and air pouches (Zhang et al., 2023)], leading to a tradeoff between simplicity and different output motions. The complexity increases in terms of operation like following a required pressure trajectory and more significantly using additional chambers (Jiao et al., 2019; Lin et al., 2020; Jiao et al., 2021). Designing a reusable and single artificial muscle architecture that is capable of alternating between different single types and sophisticated movements is therefore crucial for expanding the practical applications of pneumatic artificial muscles.

**FIGURE 1:**
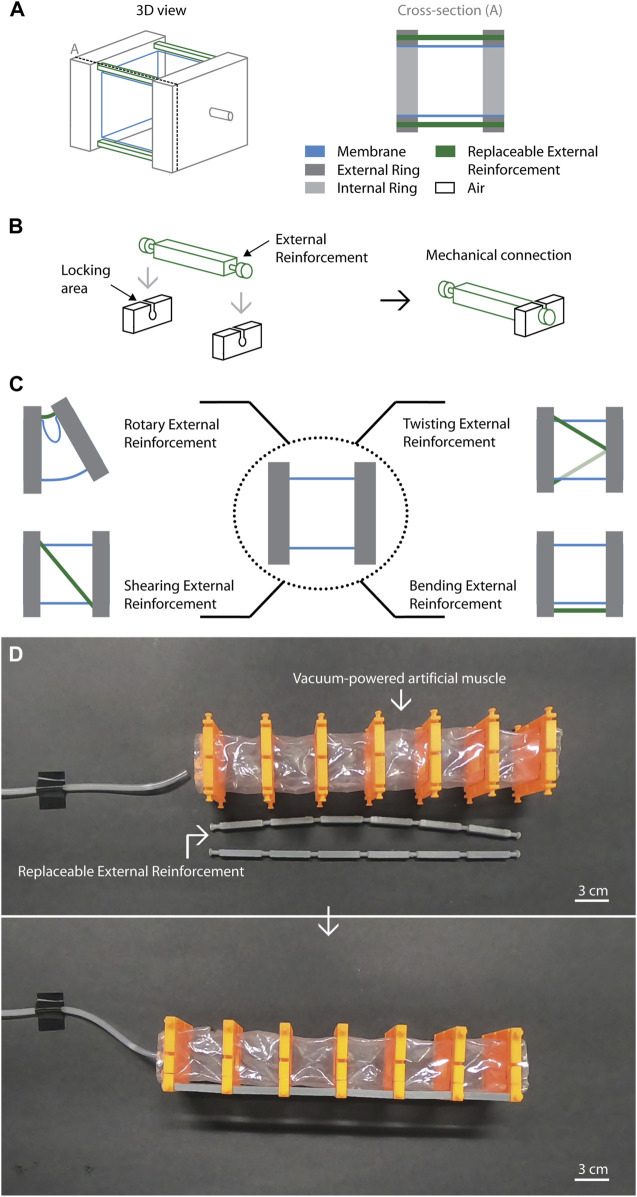
Schematics of the versatile artificial muscle. **(A)** A chamber of the artificial muscle consists of a pair of internal and external rings attached to a membrane encapsulating air. The external reinforcements can be located in the external rings of the chamber and all the chambers of the artificial muscle are actuated through a single input pressure. **(B)** The external rings have nine locking areas distributed around the lateral surface of the external ring. As the reinforcement is inserted, it encounters the locking area. **(C)** 2D Schemes of a single unit chamber using each different external reinforcement for bending, twisting, shearing and rotary motion. **(D)** The artificial muscle can be composed of several chambers and it can use a single or multiple external reinforcements that are assembled to change its output motion.

Here, we report a versatile, reusable and manually reconfigurable artificial muscle architecture capable of performing single motions (linear contraction, bending, twisting, shearing, and rotary) and sophisticated motions by combining them. The artificial muscle architecture consists of assembled square rings spaced evenly in a membrane. It includes replaceable external reinforcements that can constrain the actuator output motion according to the desired application. By replacing the reinforcements manually, a single artificial muscle can be adjusted and reused for different modes of actuation (Figure 1) expanding its versatility. We begin with a brief description of the working principle, followed by a description of the programmable motions using different external reinforcements as well as two soft robot demonstrations. We further show our experimental characterization results for a single artificial muscle in different single motions.

## 2 Materials and methods

### 2.1 Working principle

The structure of a unit chamber of the artificial muscle (AM) (Figure 1) consists of four main parts: a flexible membrane, rigid internal rings, rigid external rings, and flexible external reinforcements. In this architecture (Figure 1A), the internal and external rings act as fixed points that are equally spaced inside and outside a flexible membrane, respectively. The artificial muscle is driven by vacuum pressure that produces the shrinking of the air chamber volume as a function of the external reinforcements. Using a single input pressure, the output motion of a single unit chamber of the artificial muscle can be regulated by alternating the reinforcements, showing its capability to produce different motions such as twisting, shearing, bending and rotary motion, and being reusable in different modes of operation (Figure 1C). The external reinforcements can be assembled manually outside the air chambers of the artificial muscle using the locking areas of the external rings that work as mechanical connectors (Figure 1B). Through them, different external reinforcements in terms of materials, geometry and others can be used by an artificial muscle. Thus, the external reinforcements provide a new key feature being interchangeable and replaceable, and working as a set of conventional mechanical restrictions for changing the AM motion.

There are two main advantages of having external and interchangeable reinforcements: 1) reusing a single artificial muscle for a different task instead of building an entire new artificial muscle and 2) reducing the time required for adapting to a different task. For the output motion for an artificial muscle of one chamber, it can generate approximately 45.95° ± 0.73° in twisting motion using a pair of inclined beams, and the user can modify the twisting direction by changing the direction of the beams during the assembly. In bending motion, the output angle is 45.44° ± 0.35° and achieved by implementing a pair of straight beams. In addition to bending and twisting, the artificial muscle can perform shearing motion by attaching a pair of inclined beams in the same direction (range of motion of 26.97° ± 1.42°) and rotary motion by using a pair of curved beams (range of motion of 22.83° ± 3.61°). A detailed description of the characterization of this artificial muscle is in the results section.

Our scheme is inspired on BVA since they offer a framework that can produce large deformation and have a multiple number of air chambers. In contrast to most vacuum-powered artificial muscles limited to perform a single output motion such as BVA and FOAMs (Felt et al., 2018; Lee and Rodrigue, 2018), our architecture allows the user to manually tune the output motion of the AM after fabrication using the external reinforcements (as shown in Figure 1D and Supplementary MaterialC). In this study, we centered on flexible beams as replaceable external reinforcements for changing the output motion.

The fabrication process involves the material and components showed in Supplementary Figure S1 (see a list of them in Supplementary Table S1), and it consists of two main steps following conventional techniques: 1) the membrane preparation and 2) the artificial muscle assembly (see Supplementary Figures S2, S3 for visualizing the steps and Supplementary Material, for fabrication details). We fabricated artificial muscles with square cross-sectional areas (side length of 29 mm in the membrane) varying the number of chambers (1, 4 and 6 chambers). Note that all the reinforcements are 3D-printed using thermo-plastic polyurethane in this work.

### 2.2 Experimental methods

#### 2.2.1 Deformation characterization

The output deformation for each motion was characterized using three artificial muscles of 1 chamber and the different external reinforcements. We recorded the deformation through a camera (GoPro 7 Hero Black) at 30 FPS, and we added a mark with a marker pen in the artificial muscle to process the data in Tracker software. In the case of twisting motion, the camera provided a bottom view to register rotation. Throughout each experiment, one end of the AM was held in fixed position through a tweezer and the other end was allowed to move freely. For the actuation process, the vacuum pressure was applied from 0 to −15 kPa in steps of −1.5 kPa and was regulated using a manual vacuum regulator (IRV10A-C06LZN, SMC Pneumatics Inc.) connected to a vacuum chamber and a vacuum pump (RS-2). For each replaceable reinforcements, the experiment was conducted using three different artificial muscles. A total of 12 tests were conducted to characterize deformation. The average deformation, along with its associated error, as a function of pressure was reported in the results section.

#### 2.2.2 Lifting force characterization

To characterize the lifting force for each motion, we measured the deformation-pressure curve at different loads. In each experiment, the actuator was held vertically and connected to a set weight (55 g, 255 g and 505 g) through an inextensible cable (Kevlar thread). A pneumatic system with controllable pressure was set up, with an electronic vacuum regulator (ITV 2091-21N2BS5, SMC Pneumatics Inc.), and commanded by an Arduino Mega. In addition to that, the system included a vacuum chamber, a vacuum pump as well as a manual regulator (IRV10A-C06LZN, SMC Pneumatics Inc.) to create a constant input pressure (−50 kPa) for the electronic regulator. The applied pressure was increased from 0 to 18 kPa in steps of −1.5 kPa in each trial, and the resultant deformation was recorded through a camera while a set weight was attached to the artificial muscle (GoPro 7 Hero Black). For each set weight, the experiment was conducted three times using a different artificial muscle, and this process was repeated for each external reinforcement. In total, 36 tests were conducted. The computed average deformation-pressure curves, along with their error margins, are reported.

### 2.3 FEM modeling

A numerical model was created in ABAQUS (Dassault Systèmes) to capture the deformation of the artificial muscle in twisting motion as case of study. To reduce the computational cost and the complexity of the model, an artificial muscle of 1 chamber was employed including the membrane, the rings, and the twisting external reinforcements. The membrane was modelled using polyethylene film (Young’s Modulus: 250 MPa, thickness: 50 um) (Hartmann et al., 1987) with shell elements (S8) of size 0.5 mm, and with membrane idealization. The rings were modelled using polylactic acid with high stiffness (Young’s Modulus: 3,500 MPa, Poisson’s ratio: 0.3) with solid elements (C3D10) of thickness 0.5 mm, and one of the rings of the artificial muscle was fixed in displacement and rotation. The external reinforcements were modelled as a linear elastic material using thermo-plastic polyurethane (Young’s Modulus: 300 MPa, Poisson’s ratio: 0.3) with solid elements (C4D8) of thickness 1 mm. In the mechanical connectors of the fixed ring, the ends of the reinforcements were restricted only in displacement, whereas the other ends were free in terms of displacement and rotation in the other ring. A frictionless contact interaction was defined for all elements in simulation. Finally, one actuation step was created to apply vacuum pressure to the walls of the air chamber, compressing the reinforcements and rotating one ring gradually over time. The applied pressure was set as a ramp function to the desired value, and the dynamic explicit solver provided the dynamic response of the actuator at different vacuum pressures (e.g., 7.5 kPa corresponding to an applied pressure value in the deformation characterization). The position of three nodes were then extracted to compute the twisting angle.

## 3 Results

### 3.1 Deformation characterization

**FIGURE 2:**
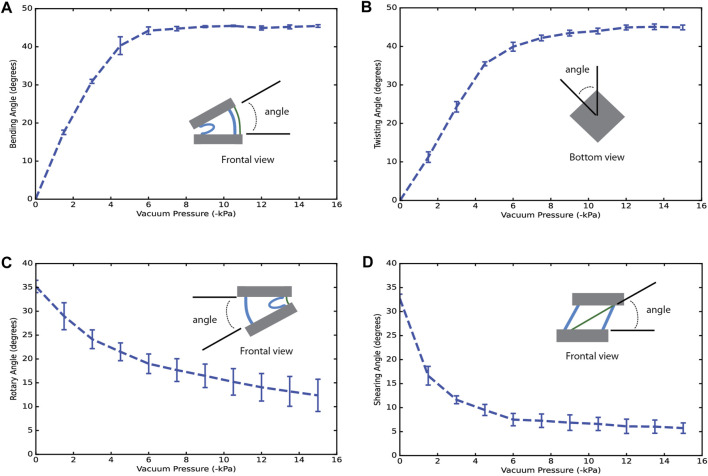
Deformation-pressure curves for the uniform motions using an artificial muscle of 1 chamber. **(A)** Angle vs. vacuum pressure using bending reinforcements. **(B)** Angle vs. vacuum pressure using twisting reinforcements. **(C)** Angle vs. vacuum pressure using rotary reinforcements. **(D)** Angle vs. vacuum pressure using shearing reinforcements. A schematic for the measured values is presented as a reference.

The results of the deformation characterization are shown in Figure 2 for each output motion. Using artificial muscles of 1 chamber with 29 mm of distance between rings, the different reinforcements were interchanged. Figure 2A shows that the output bending angle increases with the rise of the vacuum pressure, producing a maximum angle of 45.44° ± 0.35°. Using the twisting reinforcements, a similar behavior was presented, and the maximum angle measured was approximately 45.95° ± 0.73° at −15 kPa (Figure 2B). In rotary motion, the angle decreases from 35.20° ± 1.27°–12.37° ± 3.37° since the chambers shrink as the vacuum pressure increases, inducing a reduction in the initial angle. This range of motion is approximately 22.83° ± 3.61° at −15 kPa (Figure 2C). For shearing motion in Figure 2D, the angle decreases from 32.70° ± 0.92°–5.73° ± 1.08°, producing a range of motion of 26.97° ± 1.42°.

The output deformation for the different motions was limited by the stiffness and the thickness of the reinforcements since these are located between the external rings in the final collapsed states of the artificial muscles. Considering the results for an artificial muscle of 1 chamber, the bending angle or the twisting angle for a large artificial muscle of n chambers (n > 1) can be estimated. For example, an artificial muscle of 6 chambers should produce approximately 270° in bending motion, and this estimation was corroborated with our experimental results disagreeing from the expected value in 5.2% (Supplementary Figure S4A) and surpassing it in 8.5% in twisting motion (Supplementary Figure S4C), respectively.

### 3.2 Lifting force characterization

**FIGURE 3:**
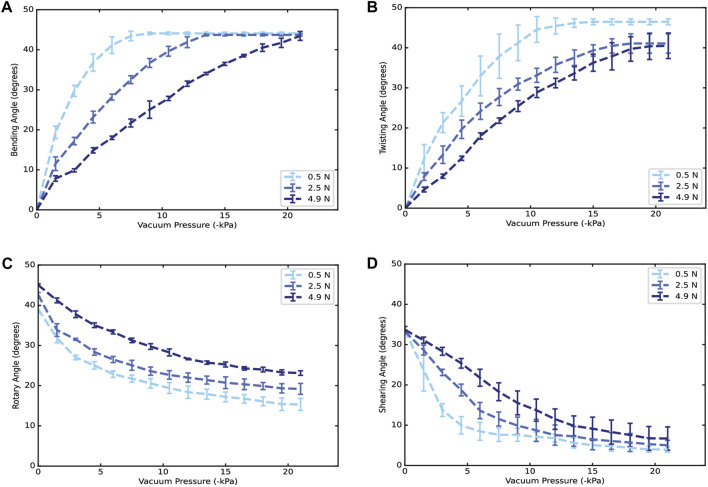
Deformation-pressure curves at different loads to show the lifting capability of a single artificial muscle. For the uniform motions using an artificial muscle of 1 chamber. **(A)** Angle-vacuum pressure curve for bending motion. **(B)** Angle vs. pressure using twisting reinforcements. **(C)** Angle vs. pressure using rotary reinforcements. **(D)** Angle vs. pressure using shearing reinforcements. The load is located in the center of the bottom ring following the reference in Figure 2.

The deformation-pressure curves in each output motion with different loads are shown in Figure 3. To characterize lifting force, three different loads were used, where we referred 0.5 N, 2.5 N and 5 N as the low, intermediate, and high load respectively. These results indicate that the load increased the required pressure to achieve the same deformation in each motion. Additionally, Figures 3A–D shows insets of the measured variable for bending, twisting, rotary, and shearing motion, respectively. In terms of reduction of performance, the most significant effect can be seen in Figure 5B for twisting motion. A reduction of 5.4° in the maximum angle was produced between the high and low load. Finally, note that the operating pressure is in the range of 0 to −21 kPa, suggesting that it is possible to increase the applied pressure to achieve maximum deformation with no loads.

### 3.3 FEM modeling

**FIGURE 4:**
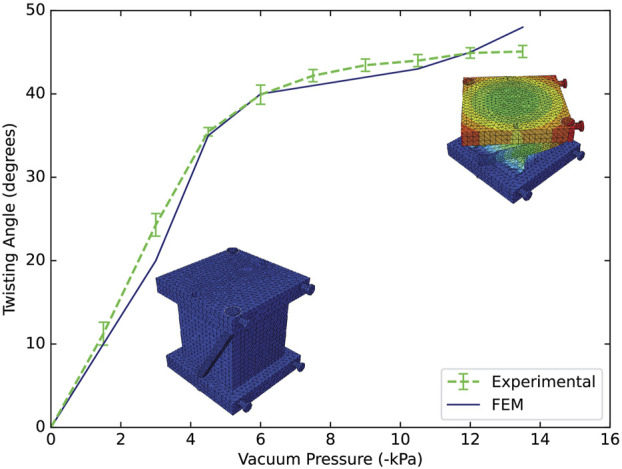
Experimental and FEM results for a single reusable artificial muscle of 1 chamber. Comparison on the artificial muscle performance using twisting reinforcements, showing series of images of the simulation at the beginning and at the end of the motion.

Figure 4 shows the comparison between the modeling and the experimental results in twisting motion for an artificial muscle of 1 chamber. The simulation predicted the shape of the curve and slightly underpredicted the output angle at the beginning of the curve and after achieving the maximum angle. Noticed that the RMSE is 1.82°, and the maximum angle error was 6.1%. Differences between model and experimental results may be caused by the fabrication process of the artificial muscle and the reinforcements. In future work, this modeling can be extended to the other uniform motions and be used for evaluating other design variations.

### 3.4 Reprogrammable motions in a large artificial muscle

#### 3.4.1 Uniform motions in a single artificial muscle

**FIGURE 5:**
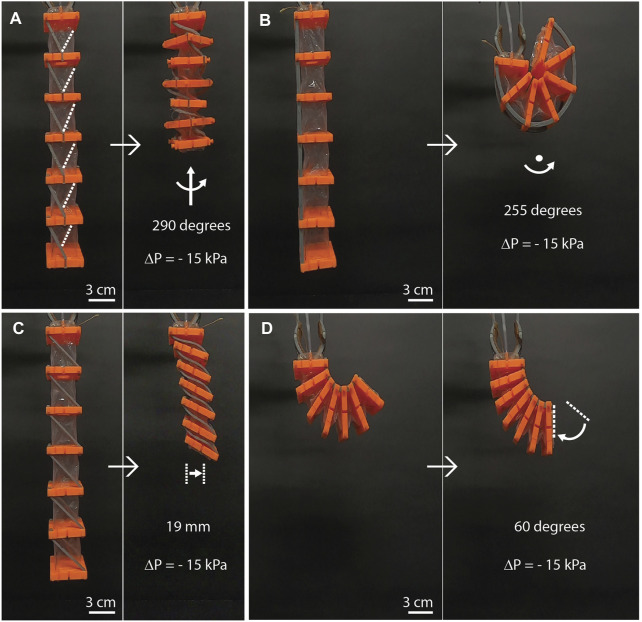
Examples of programmable and uniform motions in a single artificial muscle of 6 chambers. **(A)** A pair of inclined beams with opposite direction assembled in each chamber act as twisting reinforcements producing counter-clockwise twisting. The white dot lines indicate the inclined beams in the back of the AM. **(B)** Using a pair of straight beams to produce bending motion in all the chambers of the artificial muscle. **(C)** Using pairs of inclined beams with the same direction can produce shearing motion. **(D)** Rotary motion can be achieved by using a pair of curved beams as external reinforcements.

The large contraction is the default output motion of bellow vacuum actuators (Felt et al., 2018), this absolute contraction can be increased by adding more air chambers in the actuator as the absolute contraction of each chamber is stacked relative to one another. This large output motion can be transformed into different uniform motions using a single artificial muscle (see Figure 5; Supplementary Movie S2) and achieving large displacements and large angles in other degrees of freedom. To demonstrate achievable large deformation using the reinforcements, one artificial muscle of 6 air chambers was built, and showed the following deformations: 1) an output angle of 290° in twisting motion (Figure 5A), 2) an output angle of 255° in bending motion (Figure 5B), 3) a relative displacement of 19.5 mm relative to the axis center in shearing motion (Figure 5C) and 4) an output angle of 60° in rotary motion (Figure 5D).

#### 3.4.2 Combined motions in a single artificial muscle

**FIGURE 6:**
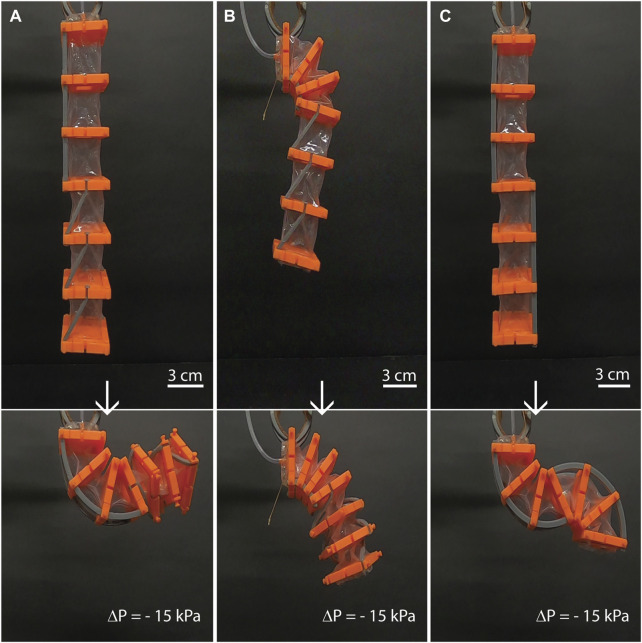
Examples of complex and versatile motions in plane and out of plane achieved by a single artificial muscle of 6 chambers. **(A)** A set of 3 chambers using pairs of inclined beams and 3 chambers using a pair of straight beams can produce a complex motion such as bending motion in plane and counter-clockwise twisting out of plane. **(B)** Rotary and twisting motion can be achieved using 3 chambers with the rotary reinforcement and 3 chambers with the twisting reinforcements. The rotary segment of the artificial muscle can lift the twisting segment. **(C)** Clockwise bending and counter-clockwise bending (an S-shape in plane) can be produced using the same type of external reinforcement locating them in different sides of the artificial muscle.

Finally, the different external reinforcements can be combined in a single artificial muscle, strategies can involve segmenting the artificial muscle in groups of chambers that perform a desired motion. A key advantage of this principle is the capability to segment and reconfigure a single unit chamber of the artificial muscle. Using the different external reinforcements enables a single artificial muscle system to produce motions in different degrees of freedom (see Figure 6; Supplementary Movie S3). Different chambers of the artificial muscle might produce bending in *X*-axis, clockwise twisting, bending in *Y*-axis, and others. For example, a single artificial muscle can achieve complex motions such as bending and twisting motion (Figure 6A) as well as rotary and twisting motion (Figure 6B) using groups of 3 chambers. Although the structural geometry of the beams is the same for the examples for simplicity, their position and direction can be tuned to achieve different complex motions such a S-shape (Figure 6C) and even produce zero net relative bending or zero net relative rotation. Thus, this architecture opens new possibilities to Vacuum-powered artificial muscles. In future work, inflatable AM can potentially be studied.

### 3.5 Soft robots driven by a single artificial muscle

As a single artificial muscle can be reused to produce complex motions. This key feature enables the actuator to drive different soft robots, offering a lightweight and reusable driven system, even without the need of additional input pressure. For example, the actuator can be segmented in different motions using external reinforcements. This strategy was employed to design two different robots such as a single scull, a boat propelled with two oars and a crawling robot using the same artificial muscle of 4 air chambers. The transition between demonstrations was recorded in Supplementary Movie S4, showing the simplicity required to change restrictions and completely change the output motion.

#### 3.5.1 Boat propelled with two oars

**FIGURE 7:**
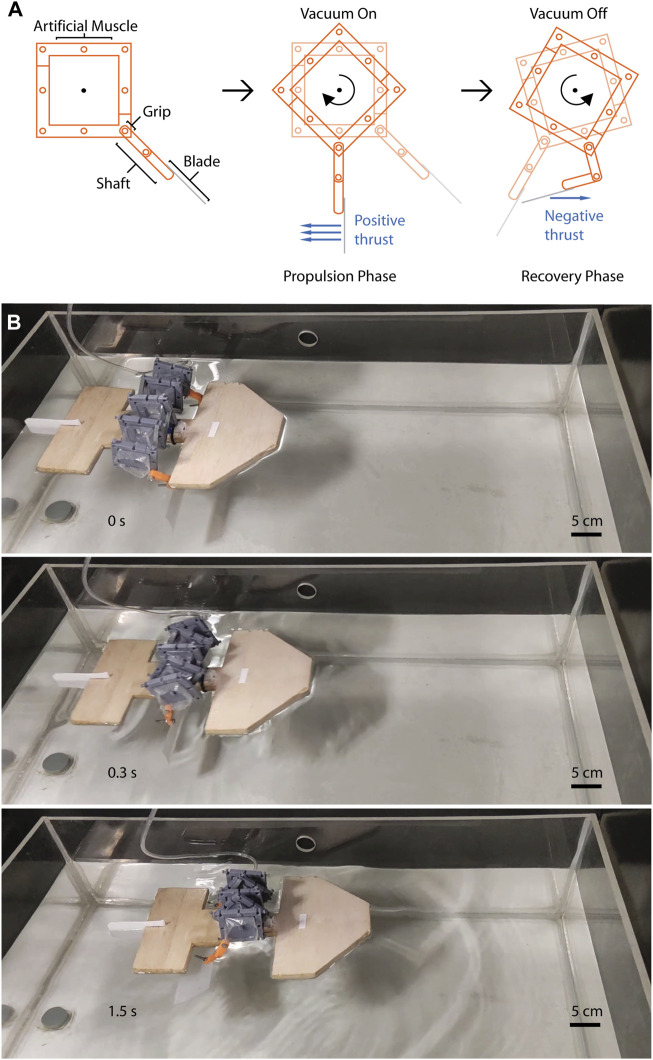
A robot using a single artificial muscle with replaceable external reinforcement as a propulsion system. **(A)** Schematics of the reusable artificial muscle using oars as additional elements to produce thrust. A shaft and a blade compose the oars added to the artificial muscle. **(B)** A single scull moves forward using a pair of oars attached to a single artificial muscle and a single input pressure. The versatility of the actuator enabled to produce clockwise twisting in its two ends (twisting reinforcements) to generate the boat thrust.

Figure 7 shows a demonstration of a single scull that can move forward using a single artificial muscle (see Movie 4, Supplementary Material). Clockwise twisting was selected to drive the boat motion, imitating the propulsion of a boat using two oars (Figure 7B). Supplementary Figure S5A shows the clockwise twisting motion prior to boat implementation in a series of images demonstrating that the two ends of an artificial muscle can produce twisting in the same direction. Otherwise, using the external reinforcements in the same direction may generate clockwise twisting and counter-clockwise twisting. The weight of the complete boat is below 150 g, including the weight of its artificial muscle of 4 chambers (<50 g). The average velocity of the boat was 0.18 BL/s and the distance travelled was approximately 330 mm. This velocity was limited due to the actuation frequency of the actuator, that was set based on the required time for the actuator to return to its relaxed state (rise time of 0.5 s and fall time of 2.5 s).

For the implementation of the boat (Supplementary Figures S5B, S5C), two oars were attached to the artificial muscle, each oar was composed of a shaft and a blade. The oars of the boat were modified to produce the stroke during actuator pressurization and fold during actuator relaxation as shown in the schematics in Figure 7A. The oars have a mechanical limit to avoid overextension during propulsion phase (positive thrust) and a revolute joint to fold to reduce the negative thrust during recovery phase. In the boat, the external rings were used as a grip point for the oars and were connected through bolts. Adding additional elements such as oars to the artificial muscle simplifies the robot design and shows that alternative mechanical connections can also be implemented. Our robot demonstrates the possibility to develop robots-based on a single artificial muscle and a single input pressure.

#### 3.5.2 Crawling robot

**FIGURE 8:**
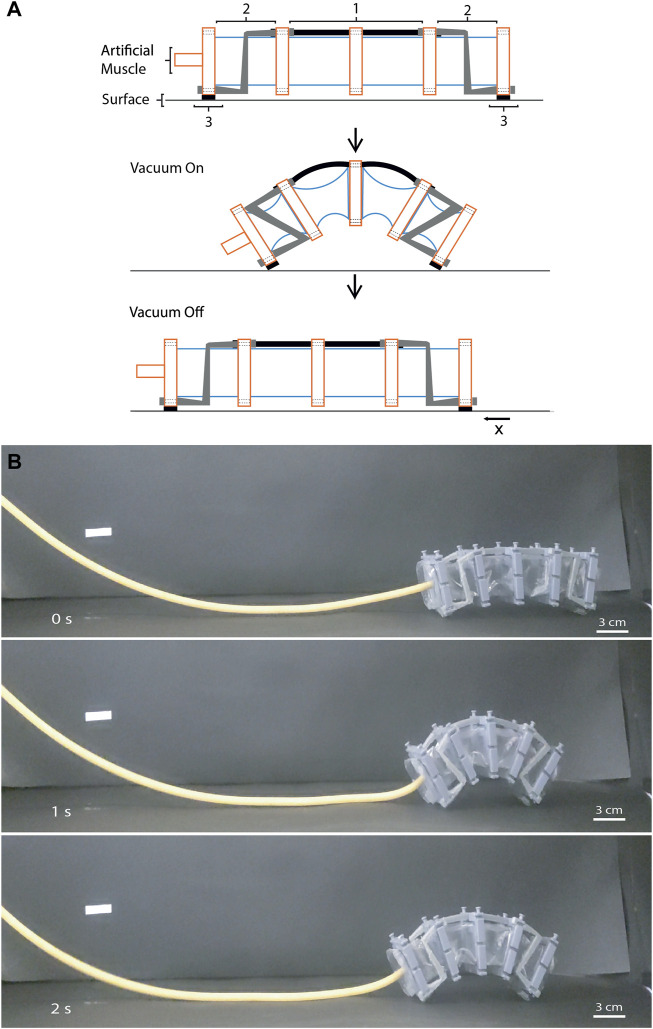
A crawling soft robot using a single artificial muscle. **(A)** Schematics of the versatile artificial muscle using different reinforcements for the crawling robot. Bending reinforcement (Thalman and Artemiadis, 2020), crawling reinforcement (Kulasekera et al., 2020) and friction reinforcement (Mendoza et al., 2021). **(B)** Series of images of the crawling robot moving forward.

This demonstration shows a crawling soft robot (Figure 8) using the same artificial muscle used in Section 3.4.1 with a different configuration of reinforcements. In this case, this soft robot uses three different reinforcements as indicated in the schematics (Figure 8A). The first is a bending reinforcement located at the top of the second and third cell that leads the ends of the AM closer to the middle position upon actuation. A crawling reinforcement was fabricated to create a C-shape and it was based on the bending and shearing reinforcements since the bending behavior reinforces the deformation of the second and third cell and the shearing behavior facilitates the displacement of the reinforcement. Additionally, this restriction acts as a stop and spring since the deformation of the chamber is stopped when the rings are in contact with the reinforcement, and the hinges of the reinforcement behave like a spring, returning the robot to an intermediate state prior to the initial position. Finally, the motion of the robot is created by using a friction reinforcement that has sandpaper to create a friction surface with the floor. One is in each end of the artificial muscle in the external rings. The reinforcement located in the right has a rougher sandpaper than the located in the left causing an asymmetric movement at the ends to generate displacement of the robot along the *x*-axis as shown in Figure 8B. The average velocity of the robot was 0.07 BL/s and the actuation frequency was 1.25 Hz. In this case, the rise time was 0.3 s and the fall time was 0.5 s. An increment of actuation frequency is expected due to spring behavior of the reinforcements in this robot in comparison to the other demonstration.

## 4 Discussion

Our architecture follows similar strategies to soft pneumatic actuators, however, most of them are limited to a fixed-shape producing a single output motion or required more than two input pressures to produce complex motions (Jiao et al., 2021). Figure 5 demonstrates the capability of external reinforcements in terms of versatility and reusability to produce single pure motions (bending, twisting or shearing) and combined motions such as bending and shearing, clockwise twisting and counter-clockwise twisting in Figure 6. Additionally, the different motions occur at low pressure (−15 kPa) using a single input pressure due to the thin film and the flexibility of the reinforcement. However, the current artificial muscle system has a fixed number of air chambers posing limitations to its application. This can be solved by building artificial muscles of one chamber, leading to the development of a modular soft robot. In contrast to other module units in soft robots that have a defined function, a module unit with replaceable reinforcement might perform different motions by changing the reinforcement thus being reusable for other applications.

### 4.1 Deformation and lifting force characterization

In comparison to other artificial muscles and soft actuators that produce a unique motion, the performance of our design is similar in terms of deformation and force. Vacuum-based soft actuators that use internal rings with a plate (Fatahillah et al., 2020) and modular unit chambers (Lin et al., 2020) to produce bending motion demonstrated similar performance to our artificial muscle of 1 chamber, and difference might be created by the dimensions and materials. Additionally, the results in shearing and rotary motion are comparable to the displacement of 5.9 mm and 22.5° presented in (Yang et al., 2017; Li et al., 2019). Finally, in the case of twisting motion positive-based fabric actuators produce twisting angle values of 80° at 100 kPa, but are limited to use long soft actuators (Nguyen and Zhang, 2020). Vacuum-based actuators can generate approximately 75° at–60 kPa (Jiao et al., 2021) or –80 kPa (Jiao et al., 2019). However, despite both vacuum actuators achieving a large twisting angle, they require at least six times –10 kPa to buckle their wall chambers during vacuum.

In terms of range of motion, stacking additional chambers in a single muscle increases the absolute deformation as shown in the Supplementary Figures S4A, S4C. As we would expect, total bending angle of 250° at −7.5 kPa for the artificial muscle of 6 chambers is similar to alternative soft actuators driven by positive pressure. However, most common soft bending actuators should undergo a high pressure (>120 kPa) (Nguyen and Zhang, 2020) and high strain (Galloway et al., 2013) to achieve a bending angle above 200°. In terms of force in a large muscle (6 chambers), the output force in vacuum-based soft actuators that perform bending (Fatahillah et al., 2020) and twisting (Jiao et al., 2019) is similar to our results (Supplementary Figures S4B, S4D). Additionally, we should note that our current external reinforcements used in the experimental characterization section have not been optimized in terms of force and deformation. For example, the current peak twisting angle is limited by the flexibility of the twisting external reinforcements that depends on their geometry and material (see Supplementary Figure S6 for details in the effect of the thickness of the reinforcements).

### 4.2 Reusability of an artificial muscle

In this study, a single artificial muscle of 4 chambers was the main actuation component in both soft robots presented. Replacing manually the reinforcements and assembling other components (oars or friction surfaces) enabled this feature. Although, alternatives approaches have been automatable instead of hand-operated, they increased the complexity of the actuation system through additional air chambers (Murali Babu et al., 2023; Zhang et al., 2023) or jamming structures (Lin et al., 2020) that can modify the fixed morphology of the actuator. This indicates a trade-off between modes of operation and simplicity in design. In future work, different smart materials and passive mechanisms can be studied to achieve a semi-automatable system, since the external reinforcements were limited to a beam shape and TPU material in this work. Additionally, other designs for mechanical connectors can be explored such as magnetic attachments and our artificial muscle also has the potential to include electronic components in the soft robots.

## 5 Conclusion

In this paper, we presented a versatile vacuum-powered artificial muscle architecture with replaceable external reinforcements. Our framework provides a modular scheme that enables to replace the reinforcements in any air chamber of the artificial muscle to produce different single motions such as twisting, bending, shearing and rotary, as well as complex motions by combining single ones. These artificial muscles have four key features for developing new soft machines: 1) a single artificial muscle can be reconfigured using replaceable external reinforcements and mechanical connectors posing the capability to be reused in different soft machines and adjust when needed, 2) single motion, combined and complexed motions can be produced by a single input pressure in a single artificial muscle simplifying the design of robots and without requiring additional systems (see Supplementary Movie S2), and 3) additional components can be added through the modular scheme as in the demonstration of the boat propelled with two oars and the crawling robot (see Movie 4, Supplementary Material) including sensors, valves or electronic components, enhancing the development of portable, autonomous soft machines. Additionally, a vacuum-powered actuator enhances the user safety shrinking during actuation up to a mechanical limit and reducing the risk of explosion, as well as offering an easy operation and low-cost platform for robots.

We provided a reusable simple architecture that can achieve large output angles in bending (<270° using 6 chambers) and twisting motion (>270° using 6 chambers). Different mechanical connectors and materials can be employed for the reinforcements, those are not limited to conventional geometries expanding the potential application of the artificial muscle for different robot designs. For example, active reinforcements based on smart materials can be explored to reshape the artificial muscle in real time. Alternatively, the artificial muscle can be used in new strategies for locomotion in swimmer and terrestrial exploration robots and in manipulation of objects to achieve a target trajectory. However, some disadvantages of this system are that there is significant risk in puncturing the membrane, shortening the cycle of actuations of the current actuator, and the modular architecture is limited to a vector with a defined number of air chambers. More importantly, the general assembly methods and the mechanical connectors are not suitable for high strength applications. We plan to address these concerns to enhance the practical engineering use of the artificial muscle in our future work.

## Data Availability

The raw data supporting the conclusion of this article will be made available by the authors, without undue reservation.
