# Supplementary material for: Versatile vacuum-powered artificial muscles through replaceable external reinforcements
Source: Front Robot AI. 2024 Jan 4;10:1289074. doi: 10.3389/frobt.2023.1289074 (PMC10794473; doi:10.3389/frobt.2023.1289074)
Supplement: Supplementary file 2 [file Table1.DOCX]

Supplementary Material

Versatile Vacuum-powered Artificial Muscles through Replaceable External Reinforcements

Mijaíl Jaén Mendoza^1^, Sergio Cancán^2^, Steve Surichaqui^3^, Esteban Centeno^1,2^, Ricardo Vilchez^1^, Katia Bertoldi^4^ and Emir A. Vela^1,5^*

*** Correspondence:** Emir A. Vela: evela@utec.edu.pe

# Supplementary Data

## Reconfigurable artificial muscle fabrication

The reconfigurable artificial muscles were fabricated through film heat sealing film, 3D printing and manual assembly. Prior fabrication, we designed the internal rings, the external rings and the end fitting rings as well as the different external reinforcements (rotary, bending, shearing reinforcements and others) using Solidworks. We employed Polylactic Acid (PLA, eSUN) and Thermoplastic Polyurethane (TPU, eSUN) in a 3D-printer Prusa MK3s for the rings and reinforcements fabrication, respectively. Supplementary Figure 1 shows the main components for the artificial muscle fabrication and Supplementary Table 1 shows a compiled list of them.

Reconfigurable artificial muscles of 6 chambers and 4 chambers were built using the materials from Supplementary Table S1 and involving two fabrication steps (Supplementary Figure 2). The first step consisted of preparing the membrane of Polyethylene (PE film, Policlick) to encapsulate the internal rings of the artificial muscle. We marked lines on the rectangular film (thickness: 0.100 mm) to guide the rings assembly and used a heat sealer (HD-300M-IR, Hurricane) for 5 seconds to obtain a cylindrical sleeve (Supplementary Figure 2a). The second step involved a general manual assembly that begins by aligning the internal rings inside the heat-sealed membrane using the lines from the first step. Following the alignment, placing the external rings outside the membrane was performed by inserting them in the lateral grooves of the internal rings (Supplementary Figure 2b). The external rings are composed of two parts that can be attached by pressure, and fixated through cyanoacrylate adhesive (Triz, Industrial Beta) in the faces in which those are connected. This process is repeated for each completed ring (assembly of internal rings and external rings) including the end fitting rings. To ensure air-tight sealing, we additionally employed epoxy syringe adhesive (5-minute Epoxy, Adhaero) in the grooves of the end fitting rings. Supplementary Figure 3 shows a reconfigurable artificial muscle of 4 chambers after fabrication and including external reinforcements for bending and shearing.

## Demonstration of a soft robot driven by a single artificial muscle

Using additional elements, an artificial muscle was capable to drive a boat as shown in Figure 4. To actuate both oars at the same time, at the beginning an artificial muscle of 6 chambers was programmed to produce only clockwise twisting using the external reinforcements and the AM was fixed in its middle ring throughout the motion testing. Supplementary Figure 5a shows this motion in a series of images demonstrating that the ends of the artificial muscle produce twisting in the same direction. Interestingly, the direction of the reinforcements of the first three chambers of the artificial muscle is the opposite of the rest of the chambers (Supplementary Figure 5a). The boat was built with an artificial muscle of 4 chambers using twisting reinforcements, a sliced balsa wood and modified oars (Supplementary Figure 5b). The oars were fabricated using PLA in a 3D-printer Prusa MK3s and modified following a similar mechanism from a buckling swimmer to produce thrust (1). The reconfigurable artificial muscle was mounted on the balsa wood similarly as it was in Supplementary Figure 5a and was expected to produce the desired motion in both ends during actuation (Supplementary Figure 5c).

## Measurement of bending angle vs. pressure in a large artificial muscle with bending reinforcements

A large artificial muscle of 6 chambers (length: 216 mm) was characterized in terms of bending angle. To characterize the output angle at different pressures, the external reinforcement for bending motion was used. Similar to the characterization of the artificial muscle with 1 chamber, we measured the bending angle through a GoPro camera at 30 FPS (GoPro 7 Hero Black) in Tracker software and used the same pneumatic circuit. Throughout the experiment, the artificial muscle was held vertically through a 3D-printed support in one end and the other was able to move freely during actuation.

We applied pressures from 0 to – 13.5 kPa in steps of -1.5 kPa. The vacuum pressure was applied to a required pressure (e.g., -9 kPa), waited for 7 s, and then decreased it to 0 kPa in each trial. The experiment was repeated three times (n = 3) for each pressure to obtain an average value. Supplementary Figure 5a shows the maximum bending angle was approximately 256.03° ± 0.23° at -13.5 kPa.

## Measurement of blocked force vs. pressure in a large artificial muscle with bending reinforcements

To measure the blocked force in bending motion, the setup consisted of two 3D-printed PLA plastics. The first 3D-printed component was for elevating and mounting the artificial muscle, and the second one for fixing a load cell (Shear load cell 5 kg). The actuator being tested was initially vertical before each experiment and was connected to the load cell using an inextensible cable (Kevlar thread) that restricted the artificial muscle tip from upward motion. We measured the output force from 0 to -18 kPa in steps of -1.5 kPa in each trial. The pneumatic system was driven by a vacuum chamber and a vacuum pump (RS-2) connected to the artificial muscle, and the pressure applied was controlled using a manual vacuum regulator (IRV10, SMC). The experiment was repeated five times (n = 5) for each pressure using different external restrictions and the force data was recorded through an Arduino Mega and the load cell amplifier (HX711, SparkFun). In this experiment (Supplementary Figure 5b), the maximum output force was approximately 4.31 N ± 0.06 N at -18 kPa.

## Measurement of twisting angle vs. pressure in a large reconfigurable artificial muscle with twisting reinforcements

We measured the twisting angle in the same artificial muscle from the bending experiments by changing the external reinforcements. The data was recorded using Matlab software, an Arduino Mega with an absolute orientation sensor (BNO055, Adafruit) located at the end of the artificial muscle. In the experimental setup, the actuator was held vertically using a fixed end with tweezers and the pressure applied was generated using a vacuum pump (RS-2) with its vacuum chamber. Additionally, the pneumatic circuit included a manual vacuum regulator (IRV10, SMC) connected to the pressure source and a solenoid valve (SMC) connected to the regulator. We applied a required pressure (e.g., -6 kPa), waited for 10 s and then decreased it to 0 kPa in each trial. The working pressure went from 0 kPa to -21 kPa. For each trial, the experiment was repeated three times (n = 3) to obtain an average angle of twisting. We tested one large artificial muscle, and the measured maximum angle was approximately 292.79° ± 0.75° at -21 kPa as shown in Supplementary Figure 5c.

## Measurement of torque vs. pressure in a large reconfigurable artificial muscle with twisting reinforcements

To obtain the torsion blocked force-pressure curve, the setup consisted of mounting the artificial muscle horizontally through a 3D-printed plastic and adding a plastic for transmitting the output force to a pair of load cells. Each load cell (shear load cell 1 kg) was fixed using a 3D-printed plastic and located horizontally to avoid undesired motion from the artificial muscle. The vacuum source was a vacuum chamber set to -50 kPa before each experiment. We applied pressure from 0 kPa to -24 kPa in steps of 1.5 kPa using a manual vacuum regulator (IRV10, SMC), and we recorded the force using load cell amplifiers (HX711, SparkFun) and an Arduino Mega. Each trial was repeated three times (n = 3) using a single artificial muscle of 6 cells. In terms of force, the torque produced by the artificial muscle with the twisting reinforcement was calculated using length half of the artificial muscle width (26 mm). Supplementary Figure 5d shows the results of this experiment. The large artificial muscle was able to produce a maximum torque of 48.43 N.mm ± 1.95 N.mm at -24 kPa.

# Supplementary Figures and Tables

## Supplementary Figures


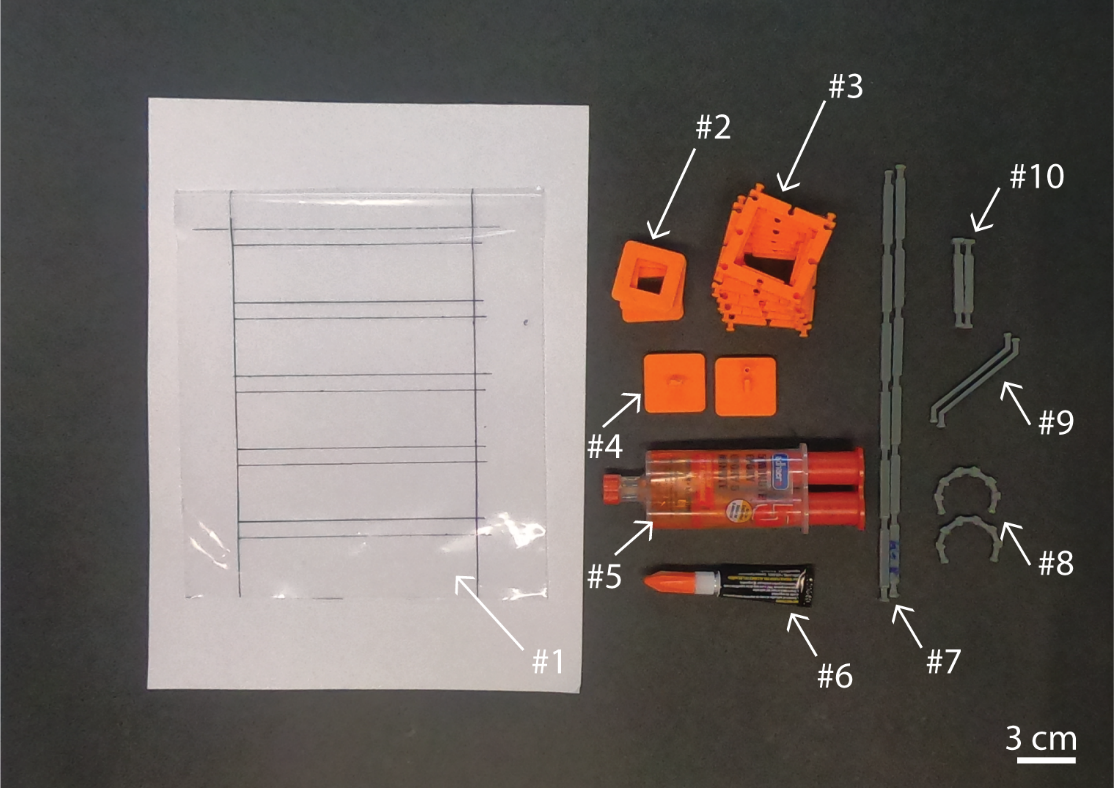


**Supplementary Figure 1.** Materials and components for building a reconfigurable artificial muscle.

**Supplementary Table 1.** List of components and materials for fabricating a reconfigurable artificial muscle.

| Item Number | Name |
| --- | --- |
| #1 | PE film |
| #2 | Internal rings |
| #3 | External rings |
| #4 | End fitting rings |
| #5 | Epoxy syringe glue |
| #6 | Cyanoacrylate glue |
| #7 | Bending external reinforcements for 6 chambers |
| #8 | Rotary external reinforcements for 1 chamber |
| #9 | Shearing external reinforcements for 1 chamber |
| #10 | Bending external reinforcements for 1 chamber |


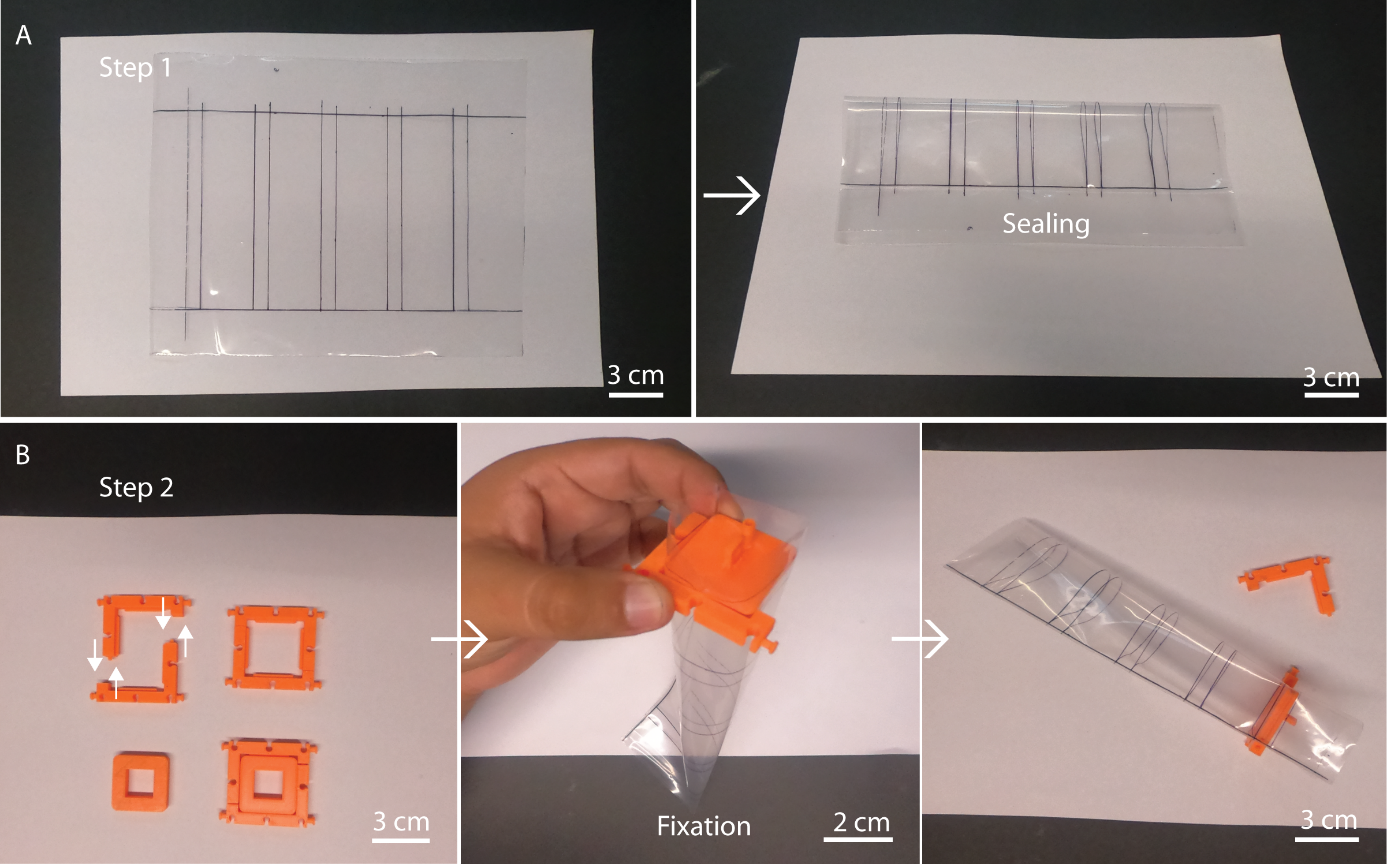


**Supplementary Figure 2.** Steps for fabricating a reconfigurable artificial muscle. A) Step 1 consists of preparing the membrane. B) Step 2 refers to the general assembly of the artificial muscle.

**
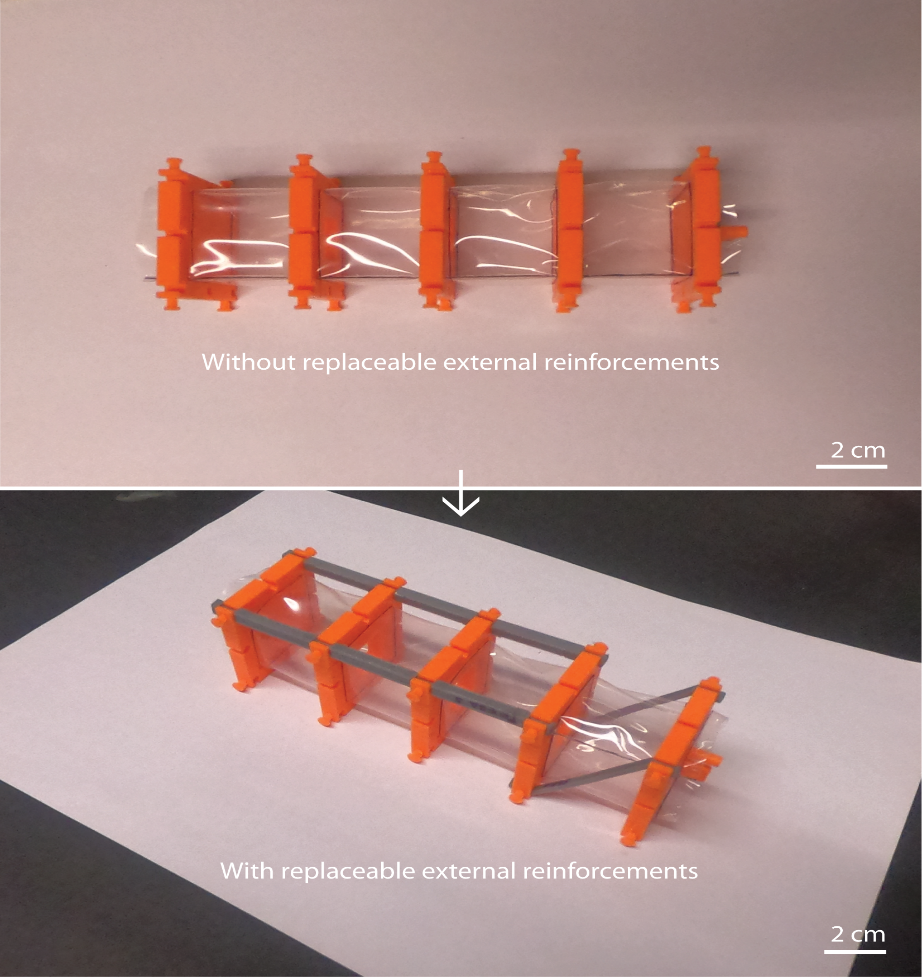
**

**Supplementary Figure 3.** A fabricated reconfigurable artificial muscle without and with replaceable external reinforcements.


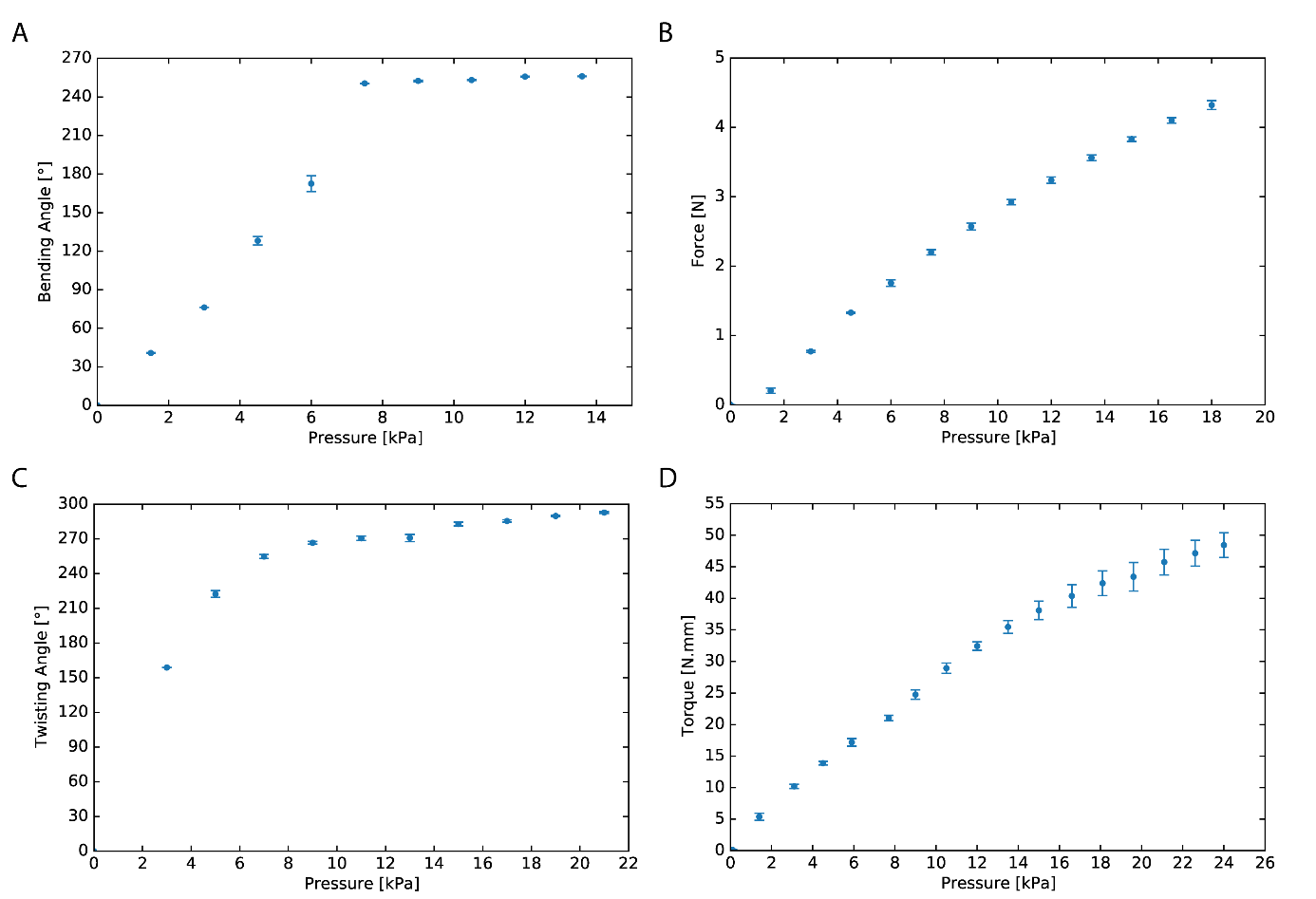


**Supplementary Figure 4.** Experimental characterization of a reconfigurable artificial muscle of 6 chambers. Using bending external reinforcements, A) angle of bending vs. pressure and B) blocked force vs. pressure. Using twisting external reinforcements, C) angle of twisting vs. pressure and D) torque vs. pressure. Note each X-axis refers to the vacuum pressure magnitude.

**
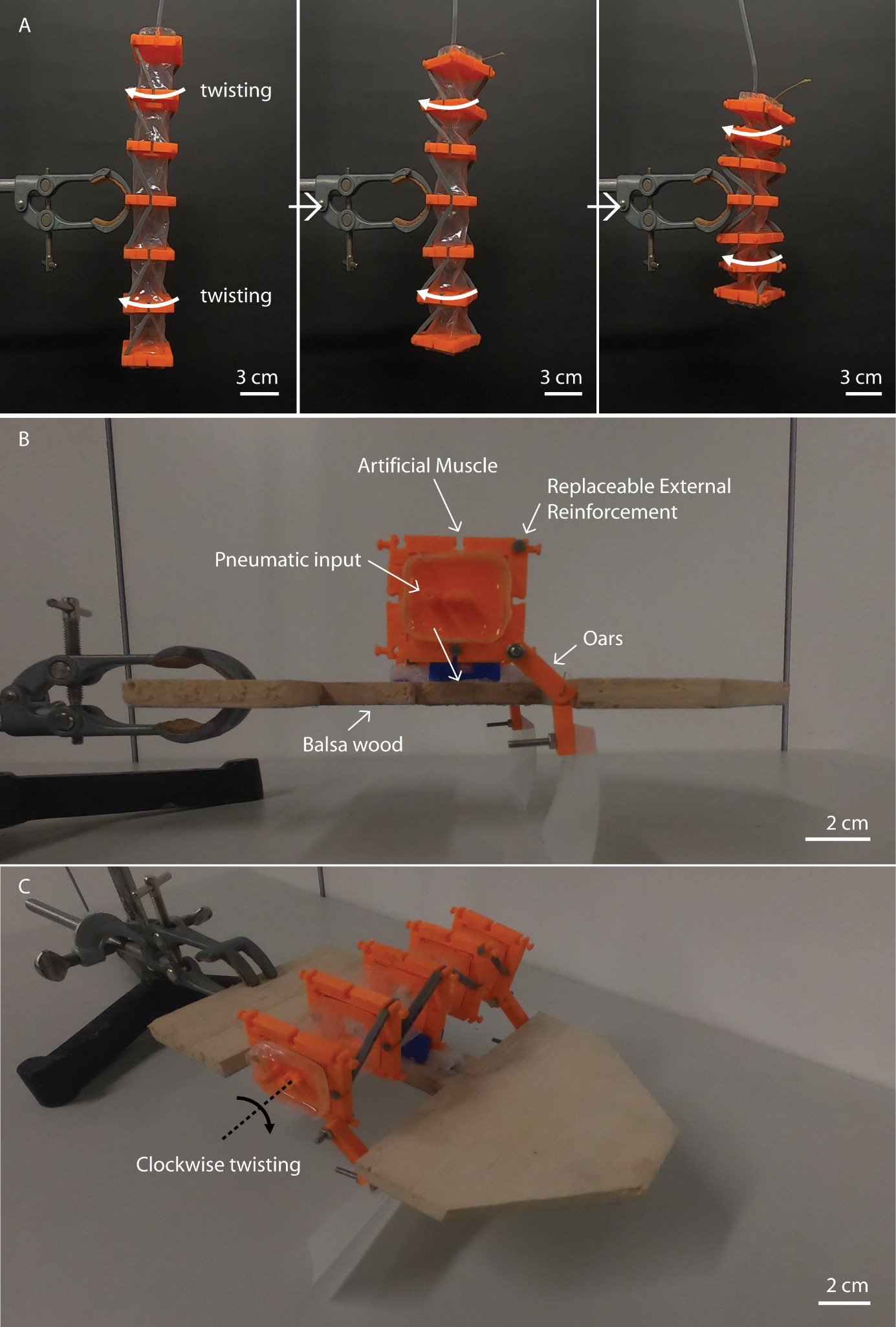
**

**Supplementary Figure 5.** Details of the soft robot driven by a single reconfigurable artificial muscle. A) A single artificial muscle fixed in its middle ring can produce clockwise twisting in both ends. B) Lateral view of the soft robot indicating its main components. C) Isometric view of the soft robot.


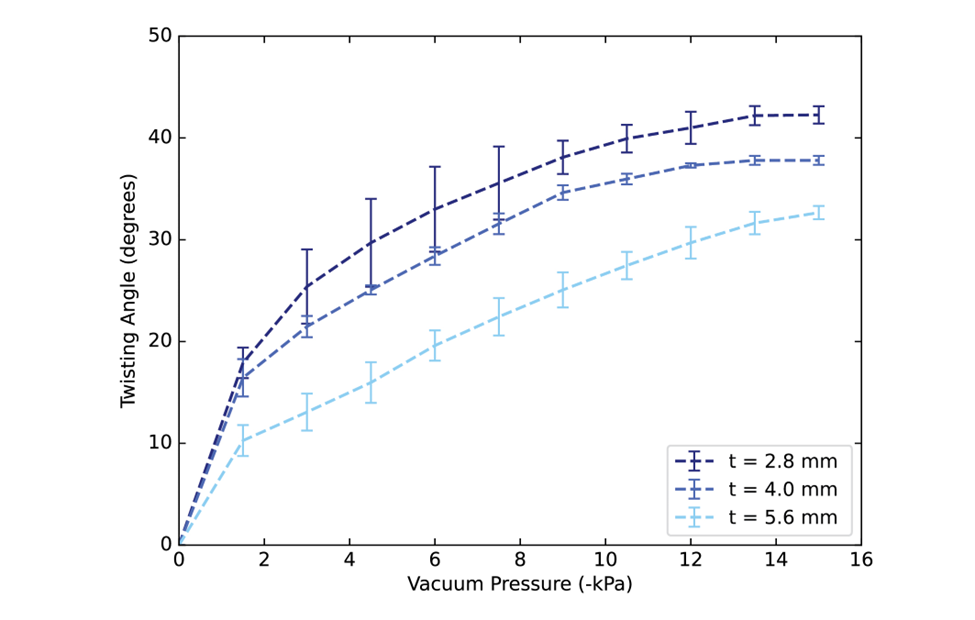


**Supplementary Figure 6.** Analysis design of the thickness of the reinforcements in the deformation in twisting motion shows a reduction in angle as the cross-sectional area increases. An artificial muscle of 1 chamber was used with reinforcements of thickness t of 2.8 mm, 4 mm, and 5.6 mm. Difference in results are caused by imperfections in the actuator fabrication.

References:

1. Yang D, Mosadegh B, Ainla A, Lee B, Khashai F, Suo Z, et al. Buckling of Elastomeric Beams Enables Actuation of Soft Machines. Advanced Materials. 2015;27(41):6323–7.

2. Hartmann B, Lee GF, Wong W. Tensile yield in polypropylene. Polym Eng Sci. 1987;27(11):823–8.
